# Rutin (Bioflavonoid) as Cell Signaling Pathway Modulator: Prospects in Treatment and Chemoprevention

**DOI:** 10.3390/ph14111069

**Published:** 2021-10-22

**Authors:** Pratibha Pandey, Fahad Khan, Huda A. Qari, Mohammad Oves

**Affiliations:** 1 Department of Biotechnology, Noida Institute of Engineering and Technology, 19, Knowledge Park-II, Institutional Area, Greater Noida 201306, India; pratibhapandey.bio@niet.co.in; 2 Department of Biological Science, Faculty of Sciences, King Abdulaziz University, Jeddah 21589, Saudi Arabia; hagari@kau.edu.sa; 3 Center of Excellence in Environmental Studies, King Abdulaziz University, Jeddah 21589, Saudi Arabia

**Keywords:** rutin, therapeutic potential, cancer, cell signaling pathways, miRNA

## Abstract

Cancer is a complex ailment orchestrated by numerous intrinsic and extrinsic pathways. Recent research has displayed a deep interest in developing plant-based cancer therapeutics for better management of the disease and limited side effects. A wide range of plant-derived compounds have been reported for their anticancer potential in the quest of finding an effective therapeutic approach. Rutin (vitamin P) is a low-molecular weight flavonoid glycoside (polyphenolic compound), abundantly present in various vegetables, fruits (especially berries and citrus fruits), and medicinal herbs. Numerous studies have delineated several pharmacological properties of rutin such as its antiprotozoal, antibacterial, anti-inflammatory, antitumor, antiviral, antiallergic, vasoactive, cytoprotective, antispasmodic, hypolipidemic, antihypertensive, and antiplatelet properties. Specifically, rutin-mediated anticancerous activities have been reported in several cancerous cell lines, but the most common scientific evidence, encompassing several molecular processes and interactions, including apoptosis pathway regulation, aberrant cell signaling pathways, and oncogenic genes, has not been thoroughly studied. In this direction, we attempted to project rutin-mediated oncogenic pathway regulation in various carcinomas. Additionally, we also incorporated advanced research that has uncovered the notable potential of rutin in the modulation of several key cellular functions via interaction with mRNAs, with major emphasis on elucidating direct miRNA targets of rutin as well as the process needed to transform these approaches for developing novel therapeutic interventions for the treatment of several cancers.

## 1. Introduction

Cancer is a multifactorial ailment with an entangled cell landscape highlighted by a chain of complex molecular mechanisms and interactions. Advancements in proteomic- and genomic-based approaches have made it possible to unveil the tumor microenvironment in order to gain a better insight into key mechanisms such as overexpression, gene suppression, altered cellular signaling pathways, genomic instability, and mutations at the genetic/epigenetic level within the genetic framework involved in cancer progression [1,2].

Accumulating evidence has led to plant extract-based formulations and compounds gaining special attention in the management of several malignancies including cancer, neurodegenerative disorders, cardiovascular diseases, and diabetes [3,4]. Further, they have displayed significant potential in modulating the expression of chief signaling pathways associated with cancer progression [5,6,7,8]. Numerous plant-based chemotherapeutic drugs have displayed significant increases in the anticancer efficacies of numerous chemotherapeutic agents, including vinblastine, doxorubicin, camptothecin, and paclitaxel [9,10,11]. Plant metabolites have displayed pleiotropic efficacies, and they target numerous cancer hallmarks such as inflammation, angiogenesis, cancer cell growth and proliferation, invasion, migration, and metastasis [12,13,14].

**Figure 1:**
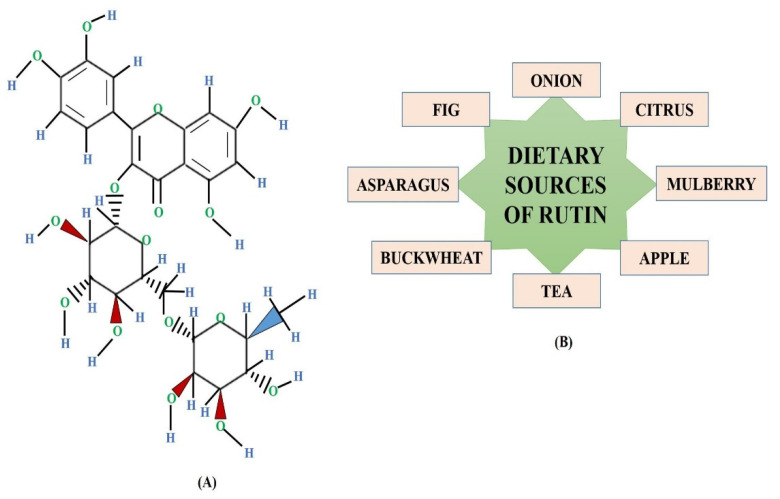
Structure and dietary sources of rutin (bioflavonoid): (**A**) structure and (**B**) dietary sources.

The name rutin (green-yellow-colored, needle-shaped crystal) originates from *Ruta graveolens* L., a plant that is rich in rutin. Rutin has also been named rutoside, vitamin P, quercetin-3-O-rutinoside, and sophorin, with the chemical formula, C_27_H_30_*O*_16_ and a molecular weight of 610.53. The natural sources of rutin are fruits, medicinal herbs, and plants (Figure 1A,B).

Rutin can be considered a safe anticancerous agent with very few or limited side effects (such as MDR (multiple drug resistance)) and limitations (high cost) in comparison to other cancer therapies including surgery, stem cell therapy, radiotherapy, chemo/immunotherapies, and photodynamic therapy [15,16,17,18,19]. This has further motivated us to comprehensively cover the therapeutic potential of rutin in tumor proliferation and invasiveness leading to apoptotic induction in cancerous cells. Rutin is a naturally occurring bioflavonoid abundantly reported in medicinal herbs, fruits, vegetables, and plant-based beverages. Research has further presented the in vitro potential of rutin in the suppression of numerous human cancers such as lung cancer, prostate cancer, colorectal cancer, breast cancer, liver cancer, glioblastoma, melanoma, osteosarcoma, ovarian cancer, leukemia, cervical cancer, and pancreatic cancer via apoptosis induction, immunity enhancement, or cell migration knockdown, which leads to a significant reduction in the motility rate of cancerous cells [20,21,22,23,24].

## 2. Anticancerous Therapeutic Potential of Rutin

Rutin has been reported to counteract numerous cancers via several mechanisms such as cell cycle arrest, inflammation, malignant cell growth inhibition, oxidative stress, apoptosis induction, and angiogenesis modulation, and all of these are mediated through the regulation of cellular signaling pathways. Several in vitro studies have reported the significant anticancerous potential of rutin via its inhibition of the proliferation of several cancer types including glioma, breast, liver, pancreas, colon, lung, prostate, skin, ovarian, and cervical cancer (Table 1).

Apoptosis has been recognized as a crucial phenomenon for the maintenance of balanced cell growth [68]. However, altered cell signaling has been widely reported with a distressed apoptotic balance, leading to cancer proliferation and invasiveness. DDR (death receptor pathway) initiation has been linked with TRAIL (TNF-related apoptosis inducing ligand) and FasL (Fas ligand) binding with transmembrane receptors. This interaction ultimately results in caspase activation that subsequently promotes apoptosis pathway (intrinsic or extrinsic) activation. In addition, apoptosome formation (associated with caspase-9, cytochrome-C, and apoptotic protease-activating factor) triggers caspase-3 activation, leading to cell death. Several in vitro and in vivo studies have elaborated the chemopreventive potential of rutin against several cancer types (Table 2).

## 3. Interaction of Rutin with Numerous Molecular Signaling Pathways

Cancer cells rapidly develop drug resistance against various therapeutic approaches which presents a major hindrance in the cancer management research of several pharmacologists and molecular biologists. This has further motivated researchers to develop drugs with limited cytotoxicity and high specificity. The following subsection explains rutin’s interactions with various molecular pathways.

### 3.1. Rutin’s Involvement in Modulation of Akt/PI3K/mTOR Signaling Pathway

Gene expression, translation, and transcription, and cell growth and proliferation are some of the major roles controlled by the Akt/PI3K/mTOR signaling pathway. Several reports have established the association between abrogation in this signaling cascade and the progression of numerous carcinomas by triggering tumor growth, invasion, and metastasis [81,82]. In brief, PI3K is found downstream of tyrosine kinase growth receptors, due to which mutations in the Akt protein can inversely affect effector proteins and downstream signaling, thereby stimulating cancer cell growth and development [83]. Rutin has been found to employ its effect on the AkT/PI3K/mTOR pathway by modulating the expression of several key proteins, including AkT, PTEN, ERK, and others [84,85,86]. Later, it was further reported that rutin blocks the cross-talk between AkT and PI3K by blocking PI3K activity. Rutin treatment has also resulted in the attenuation of H_2_O_2_-induced oxidative damage and in apoptotic induction in Leydig cells via PI3K/Akt signaling pathways [87]. In addition, rutin prevents AkT phosphorylation by disturbing the PI3K-ATP binding domain and mTORC2 complex. Rutin mediated apoptotic induction via modulating Bcl-2, Bax, and caspase expression levels and also induced antioxidant activity by increasing antioxidants levels [88]. Rutin also deregulated the expression of numerous molecules, including GSK-3 (Glycogen synthase kinase-3). Further, rutin promoted TNF-α-induced apoptosis in A549 human lung cancer cells by modulating the expression level of the GSK-3β protein [89]. Rutin has also prevented GSK-3β phosphorylation by regulating PI3K expression and has displayed protection against γ-radiation or acrylamide-induced neurotoxicity through activation of the PI3K/AKT pathway by deregulating phosphorylation. Rutin prevents GSK-3β (AkT target) phosphorylation via PI3K inhibition [90]. Additionally, rutin has also been reported to increase the expression level of tumor suppressor proteins including FOXO3a, p21, KIP1, and CIP/WAF. The increased expression levels of these reported tumor suppressor proteins further promoted growth arrest in cancer cells. Rutin treatment prevented cisplatin (CP)-induced ovarian damage by regulating FOXO3a and PTEN phosphorylation and antioxidant activity in a mouse model [91]. Rutin has also been associated with the regulation of mTOR activity by modulating TSC2 expression [92]. Rutin-mediated protein kinase β activation promotes AMPK activation which results in TSC2 phosphorylation and inhibition of mTOR activity [93,94,95]. Altogether, these findings strongly suggest a reciprocal feedback mechanism which involves mTOR and AMPK in cancer cells and could be targeted for elucidating and developing potent therapeutics.

### 3.2. Rutin’s Involvement in Modulation of STAT Signaling

**Figure 2:**
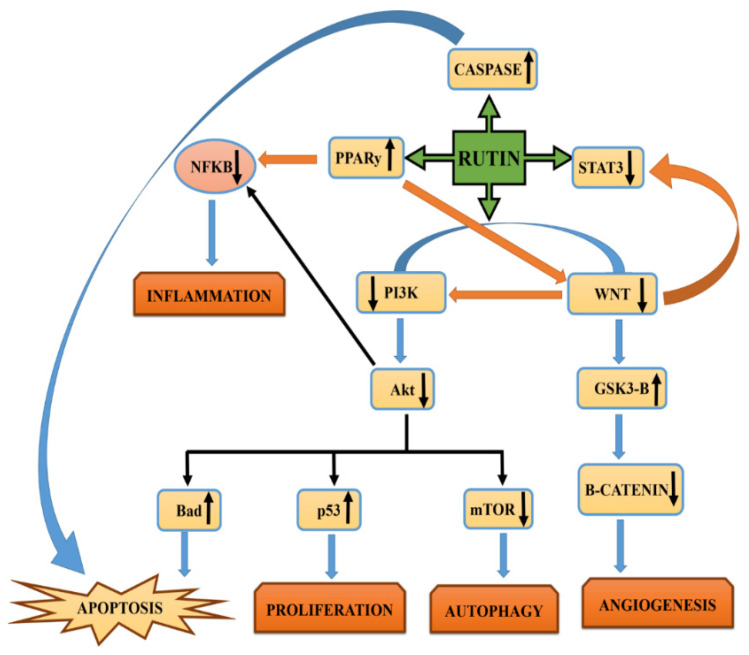
Mechanism associated with the anticancer potential of rutin via the targeting of numerous molecular signaling pathways.

The STAT pathway is an evolutionarily conserved signaling pathway that is involved in the regulation of numerous cell processes including inflammation, immune cell development, migration, cell survival, apoptosis, cell homeostasis, and cell proliferation. Aberrant STAT signaling has been recognized as a hallmark of numerous cancers [96,97,98,99]. Rutin has been exploited for its potential role in inhibiting cancer cell growth and metastasis by inhibiting this signaling pathway. Rutin modulates this cellular pathway via the repression of SRC (kinases) phosphorylation, thereby inhibiting STAT 3 (signal transducers and activators of transcription 3) activation which further blocks the translocation of STAT to the nucleus [100,101]. STAT 3 has also been associated with the regulation of MMPs (membrane metalloproteases), VEGF, and TWIST1 which are crucial for cancer cell migration, proliferation, invasion, and angiogenesis. Rutin blocks the activation of TWIST1, MMPs, and VEGF via the inhibition of STAT3 phosphorylation [102,103]. Another study also reported that rutin treatment blocked UVB-induced STAT 3 activation via the inhibition of Tyr705 phosphorylation which explains the usefulness of rutin in preventing skin cancer [103]. Several studies have evidenced its potential anticancerous role in regulating the expression of key signaling components (STAT5, STAT3, and JAK2) in numerous carcinomas, thereby preventing cancer cell growth and differentiation (Figure 2).

### 3.3. Rutin’s Involvement in Modulation of Wnt/β Catenin Signaling

Wnt/β catenin signaling contributes a crucial role in differentiation, organogenesis, cell migration, tissue homeostasis, and neuronal patterning. Modulated Wnt/β catenin signaling has been associated with the invasiveness and progression of several carcinomas [104,105,106]. Rutin has also been reported to induce variation in the expression level of downstream effectors of Wnt/β catenin signaling, including AXIN2, c-MYC, and cyclin D1 [107]. Further research studies have also revealed that rutin prevents the stabilization and accumulation of β-catenin in the cytoplasm and also blocks its translocation in the nucleus via the repression of the PI3K/AkT/mTOR pathway [68]. From these research findings, it can be concluded that rutin can be considered a potent therapeutic option for the management of numerous carcinomas.

### 3.4. Rutin’s Involvement in Modulation of MAPK Signaling

The MAPK family consists of three major classes of activated kinases including ERK/MAPK, p38 kinase, and c-JUN/SAPK that play a crucial role in cell proliferation, invasion, homeostasis, and differentiation and cell death. Overexpression of numerous ERK protein family members has been exclusively reported in the modulation of the MAPK-ERK pathway [108]. In numerous carcinomas, ERK signaling is activated via mutation in either the tyrosine kinase receptor or the kinase genes RAS and RAF. Rutin has presented significant antiproliferative effects in numerous cancer cell lines by modulating the expression of DR4/DR5, AkT, ERK, and NF-kB. Rutin exposure has promoted TRAIL-mediated apoptosis in numerous carcinomas such as lung cancer [109,110,111,112]. Additionally, rutin has been utilized to suppress the growth, invasion, proliferation, and metastasis of several melanoma cells. Specifically, in A375 and C8161 melanoma cell lines, rutin administration promoted growth arrest via the downregulation of p-AkT, p-ERK1/2, and p-mTOR. Rutin treatment has significantly downregulated the expression level of p-ERK1/2 (phospho-extracellular signal-regulated kinase 1/2) in glioma cells [113]. Other studies have also reported that rutin treatment leads to a significant reduction in the expression level of p38 MAPK in lung cancer (A549) cells. Moreover, rutin has strong potential to activate p38 MAPK in colon cancer cells (HT-29), either alone or in combination with silibinin [112]. Conclusively, these experimental findings shed enormous light on the fact that rutin can induce apoptosis via the suppression of the aberrant MAPK/ERK signaling pathway.

### 3.5. Rutin’s Targeting of Apoptotic Pathways and Autophagy Signaling Molecules

Rutin has presented potent efficacy in apoptosis induction in cancer cells. Rutin elicited the intrinsic apoptosis pathway in neuroblastoma cells as shown by the reduced Bcl2 protein and Bcl2/Bax ratio [114]. Likewise, in HCT (colon cancer) cells, rutin treatment induced caspase-3 activation [43]. Furthermore, rutin has been shown to activate both the intrinsic and extrinsic apoptotic pathways in colon cancer cells (HT-29) by upregulating caspases and Bax, and downregulating Bcl-2 levels [110]. This evidence strongly supports the apoptosis-inducing potential of rutin in cancer cells via the induction of both the intrinsic (mitochondria-mediated) and extrinsic (death receptor-mediated) apoptotic pathways.

Rutin exposure to cancer cells can also induce apoptosis via p53 activation [33,54,110]. Rutin treatment can also lead to an increase in the PTEN (tumor suppressor) mRNA expression level, resulting in apoptosis-mediated growth arrest in breast cancer cells [33,115]. ER targeting has also emerged as one of the promising approaches for cancer prevention and treatment [116]. A study conducted by Nasri Nasrabadi [44] et al. identified altered molecular signaling pathways and differentially expressed genes in rutin-treated colorectal cancer cell lines. ROS (reactive oxygen species), generated during metabolism, are also associated with numerous physiological functions. The balance between ROS production and their elimination via the antioxidant defense system is properly regulated in normal cells, whereas ROS homeostasis is dysregulated in cancerous cells, leading to elevated ROS levels. ROS have also been explored as a potent therapeutic tool for cancer management because elevated ROS generation could trigger apoptosis in cancer cells [117]. Rutin treatment has induced significant apoptosis by activating ROS-dependent apoptosis pathways in numerous cancer cells including MCF-7, HepG2, LoVo, HeLa, and C33A cancer cells [35,40].

Deregulated autophagy (catabolic process) has also been crucially associated with several cancer types. Signaling molecules such as ATGs (autophagy-related genes), which are mainly involved in autophagy, include ATG6, ATG5, ATG12, and LC3 (microtubule associated protein 1 A/1B-light chain 3). Several studies have projected that autophagy greatly contributes to the inhibition of cancer cell growth. Further, rutin treatment has induced autophagy in several cancer cells such as A549, THP1, and CA9-22. Rutin treatment has also resulted in an enhanced expression level of Beclin1 and has triggered the formation of the ATG5/12 complex. LC3-II (hallmark of autophagy) activation was also observed in rutin-treated cancer cells, thereby leading to autophagy-mediated cancer cell death [57].

## 4. Rutin and miRNA (microRNAs) Interplay: Potent Approach in Cancer Management

MicroRNAs (small molecules with a size range of 10–25 bp) have received profound attention in cancer management therapies because of their potent involvement in gene transcription and translation [118]. Several studies have reported that dysregulated microRNAs (miRNA) make major contributions to cancer progression and thus they can be considered an effective target in cancer therapies. This section focuses on revealing the interplay between rutin and miRNAs, and how rutin treatment modulates the expression of various tumor suppressor and oncogenic miRNAs in cancer. Recently, it has come to light that rutin restrains the proliferation of mouse breast cancer cells via the regulation of the miR-129-1-3p/Ca2+ signaling pathway, thereby revealing its potential as a strong drug candidate for the inhibition of tumor growth. Rutin, in combination with various miRNA mimics and inhibitors, has aided in cell apoptosis or growth arrest in numerous cancer cells [119,120]. Rutin has also shown significant inhibition of myocardial oxidative insults by adjusting ROS (reactive oxygen species) levels. This further explains the protective effect of rutin on THP cardiotoxicity via the regulation of JunD gene expression by miR-125b-1-3p, which reveals the protective efficacy of rutin on THP-induced cardiotoxicity and provides a strong base for the utilization of rutin as a potent protective candidate against THP cardiotoxicity. Thus, it may be concluded that rutin could modulate several cancer-relevant miRNAs such as miR-155, let-7, miR-146a, and miR-21, thereby potentially inhibiting cancer development and progression [121,122,123,124]. However, more experimental research is still needed to study how rutin modulates this complex and broad range of both tumor suppressor and oncogenic miRNAs. Therefore, future studies should emphasize elucidating direct miRNA targets of rutin as well the process needed in transforming these approaches for developing novel therapeutic interventions for numerous cancers.

## 5. Conclusions

In several natural products, rutin contributes to potent biological activities including anticancerous effects. Rutin has been shown to utilize numerous mechanisms to obstruct cancer initiation and progression by modulating several deregulated signaling pathways involved in apoptosis, inflammation, angiogenesis, and autophagy. Specifically, the anticancer potential of rutin has been linked to the regulation of multiple signaling pathways, including NF-κB, PI3K/Akt/mTOR, Nrf2, ERK, JNK, and p38 MAPK. This bioactive plant-derived compound significantly interferes with numerous intracellular signaling molecules, such as TNF-α, Bax, ILs, Beclin, VEGF, Bcl-2, and caspases. Extensive in vitro and in vivo studies have clearly revealed therapeutic targets of rutin such as Bcl-2, p53, caspases, Bax, NF-κB, TNF-α, Akt, and GSH. Rutin has shown tremendous anticancer potential against a range of cancer cell lines including glioblastoma, breast cancer, lung adenocarcinoma, prostate cancer, cervical cancer, gastric cancer, leukemia, hepatocellular carcinoma, and colon cancer cell lines. Despite several preclinical mechanistic reports on the anticancer efficacies of rutin, the lack of well-framed clinical trials on the safety and therapeutic potential of rutin increases the need for more potent clinical studies. Further, more elaborative studies (concerning engineering methods) are also needed to achieve a better targeted drug delivery approach for cancer management. Altogether, researchers should focus their study towards elucidating the novel molecular targets of rutin and the associated mechanism by which rutin mediates cancer cell growth arrest via molecular cross-talks and signaling cascades. Overall, rutin has enormous medicinal potential and could be employed as a potent therapeutic agent through extensive investigation into its potential to modulate numerous cell signaling pathways and apoptosis pathways involved in cancer progression.

## Figures and Tables

**Table 1:** In vitro antitumor efficacy of rutin and its mechanisms of action.

Cancer	Cell Lines	Doses	Anticancer Mechanism	Molecular Targets	References
Lung cancer	A549	20–560 µM	Cell growth, invasion, and adhesion inhibition; apoptosis and autophagy induction	p38, NF-κB, TNF-α, GSK-3b, Beclin-1	[25,26,27,28,29]
	GLC4 cells	4 µM	Cell growth inhibition		[30]
Breast Cancer	MDA-MB-231 cells	80.0–640 µg/mL	Cell growth, invasion, metastasis, and adhesion inhibition; apoptosis and induction	c-Met kinase	[31,32]
	MCF-7 cells	19.4–46.1 µM	Cell growth inhibition via apoptotic induction	p53, PTEN, p21; Cyclin B, caspase 3/7, ROS	[33,34,35,36]
Cervical cancer	HeLa cells	30–265 µg/mL	Cell growth inhibition via apoptotic induction	ROS, caspase-3, E6, E7	[37,38,39]
	C33A cells	120 µM	Cell growth inhibition via apoptotic induction	ROS mediated	[40]
Colorectal cancer	HT-29 cells	100–300 µM	Cell growth inhibition via apoptotic induction	Bax, Bcl2, p53 caspases-3, -8, and-9, PARP, NF-κB, IKK-a and IKK-b, p38, and MK-2	[27,41,42]
	Caco-2 cells	711 µM	Cell growth inhibition	Superoxide	[27]
	LoVo cells	29 µM	Cell growth inhibition via apoptotic induction and cell cycle arrest	ROS	[35]
	HCT 116 cells		Cell growth inhibition via apoptotic induction	Caspase-3	[43]
	SW480 cells	600 mM	Cell growth inhibition	Cancer cell metabolism	[37,44]
Prostate cancer	LNCaP cells	75.0 mM	Cell growth inhibition via apoptotic induction	-	[45]
	PC-3 cells	91 µg/mL	Cell growth inhibition	-	[46]
Pancreatic cancer	PANC-1 cells	26 µg/mL	Cell growth inhibition via apoptotic induction	Caspase-3/7	[47]
Liver cancer	Hep G2 cells	10–200 µM	Cell growth inhibition via apoptotic induction	-	[36,47,48,49,50,51]
	Murine HTC cells	810 µM	Cell growth inhibition	-	[52]
Neuroblastoma	LAN-5 cells	25–100 µg/mL	Cell growth, invasion, and adhesion inhibition	MYCN, Bax, Bcl2, TNFa	[53]
	Neuro-2a cells	24 µM	Cell growth inhibition	-	[54]
	SK-N-SH cells	36 µM	Cell growth inhibition	-	[54]
Melanoma	SK-MEL-28	40 µM	Cell growth inhibition via apoptotic induction	GSH, ROS, MMP	[55]
Nasopharyngeal carcinoma	CNE-2 cells	5–80 mg/L	Cell growth inhibition	-	[56]
Oral cancer	CA9-22 cells	20–40 µM	Autophagy induction	NF-κB, ATG5/12 conjugation, LC3-II, Beclin-1, TNF-alpha	[57]
	KB cells	167 µg/mL	Cell growth inhibition	-	[58]
Ovarian cancer	OVCAR-3	-	Cell growth and VEGF inhibition	-	[59]
Renal cancer	786-O	45.2 µM	Cell growth inhibition	-	[60]
Gastric cancer	SGC-7901	300 µM	Cell growth inhibition via apoptotic induction	p38 MAPK pathway	[61]
Glioma	GL-15 cells	50–100 µM	Cell growth inhibition via apoptotic induction	p-ERK1/2	[62]
	CHME cells	15 µM	Cell growth inhibition via apoptotic induction	p53, Bax, Bcl2, caspase-3/-9	[54]
	LN-229 cells	22 µM	Cell growth inhibition	-	[54]
Leukemia	U-937 cells	9.6 µg/mL	Cell growth inhibition	-	[63]
	K562 cells	98.56 µg/mL	Cell growth inhibition via apoptotic induction	-	[64,65]
	ARH-77 cells	50–200 µM	Cell growth inhibition via mitochondrial and lysosomal activities	-	[66]
	Leukocytes	1.50 µg/mL	Cell growth inhibition	-	[67]
	U937 cells	80 µg/mL	Cell growth inhibition via apoptotic induction	GSK-3β	[68]
	THP-1 cell-derived macrophages	20–40 µM	Autophagy induction	NF-κB, ATG5/12 conjugation, LC3-II, Beclin-1	[69]
	Leukemia stem cells (CD123+/ CD34+/CD38+)	160 µg/mL	Cell growth inhibition via apoptotic induction	GSK-3β	[68]

Abbreviations: Atg5/12, autophagy related 5/12; Bax, Bcl-2 associated X protein; Bcl-2, B cell lymphoma 2; GSK-3β, glycogen synthase kinase 3 beta; LC3-II, light chain 3; PARP, poly (ADP ribose) polymerase; IKK, IκB kinase; MAPK, mitogen-activated protein kinase; MMP, mitochondrial membrane potential; N-myc proto-oncogene protein; NF-κB, nuclear factor kappa; B ROS, reactive oxygen species; TNF-α, tumor necrosis factor alpha.

**Table 2:** In vivo antitumor efficacy of rutin and its mechanisms of action.

Cancer Model	Cell Lines	Doses/Treatment	Anticancer Mechanism	Molecular Targets	References
Cervical cancer	Human papillomavirus type 16 (HPV16)-transgenic mice	24 weeks	Tumor growth inhibition	COX-2	[70]
	HeLa cells induced cervical cancer (i.p.) in female Wistar albino rats	50 mg/kg and 70 mg/kg rutin for 45 days	Tumor growth inhibition	Modulation of hematological parameters and lipid peroxidation	[71]
Leukemia	Human leukemia HL-60 cells (s.c.) inboth flanks of female BALB/ cnu/nu mice	120 mg/kg rutin once every four days	Tumor growth inhibition	-	[72]
	Murine leukemia WEHI-3 cells (i.p.) in male BALB/c mice	6 mg/kg and 12 mg/kg rutin for up to 3 weeks orally	Tumor growth inhibition	Modulation of whole blood cell surface markers	[73]
	Human leukemic U-937 cells in male CD1 nu/nu nude mice and CD-1 mice	5, 10, and 15 mg/kg for 9 days orally	Tumor growth inhibition	-	[74]
Breast cancer	MDA-MB-231/GFP cells induced breast cancer in female athymic Foxn1nu/Foxn1þ mice	30.0 mg/kg rutin three times a week	Reduction in tumor growth	ROS, caspase-3, E6, E7	[75]
Prostate cancer	PC-3-luc cells induced prostate cancer in male nude BALB/c mice	100 mg/kg rutin daily for 4 weeks orally	Tumor growth inhibition	-	[76]
Lung cancer	B16F10 melanoma cells induced lung cancer in female Swiss albino mice	0.2% *w*/*v* rutin for 21 days orally	Lung metastasis inhibition	Decrease in lung tumor nodules and invasion index	[77]
Colon cancer	SW480 colon cancer cells induced colon cancer in nu/nu mice	1, 10, and 20 mg/kg rutin daily for 32 days i.p.	Tumor growth and angiogenesis inhibition	VEGF	[37]
Glioblastoma	U87 glioblastoma cells induced cancer in BALB/c athymic mice	20 mg/kg rutin thrice a week for two weeks	Tumor growth inhibition via apoptotic induction	Decrease in autophagy and JNK expression	[78]
Liver cancer	DEN induced hepatocellular carcinoma in Wistar rats	50 mg/kg rutin for 16 weeks orally	Inhibition of cell proliferation	Decrease in hepatocellular marker enzymes and tumor invasion	[79]
	Aflatoxin B1 and N-nitrosodimethylamine induced hepatocellular carcinoma in Wistar rats	1 and 10 mg/100 g rutin for 2 weeks orally	Protection from carcinogenesis by enzyme modulation	Decrease in PARP, DNA ligase, and polymerase beta	[80]

Abbreviations: HPV, human papilloma virus; COX-2, cyclooxygenase-2; I.P., intraperitoneal; JNK, c-Jun N-terminal kinase; PARP, poly-ADP ribose polymerase; VEGF, vascular endothelial growth factor; ROS, reactive oxygen species.

## Data Availability

Data sharing not applicable.
